# Supplementary material for: Shedding light on dark genes: enhanced targeted resequencing by optimizing the combination of enrichment technology and DNA fragment length
Source: Sci Rep. 2020 Jun 10;10:9424. doi: 10.1038/s41598-020-66331-z (PMC7287100; doi:10.1038/s41598-020-66331-z)
Supplement: Supplementary file 1 — Supplementary information. [file 41598_2020_66331_MOESM1_ESM.pdf]

# Shedding light on dark genes: enhanced targeted resequencing by optimizing the combination of enrichment technology and DNA fragment length

**Barbara Iadarola[1,2+], Luciano Xumerle[1,2+], Denise Lavezzari[1], Marta Paterno[1], Luca Marcolungo[1], Cristina Beltrami[1], Elisabetta Fortunati[1], Davide Mei[3], Annalisa Vetro[3], Renzo Guerrini[3], Elena Parrini[3], Marzia Rossato[1] and Massimo Delledonne[1*]**

[1] Department of Biotechnology, University of Verona, Strada Le Grazie 15, 37134, Verona, Italy; barbara.iadarola@univr.it; denise.lavezzari@univr.it; marta.paterno@univr.it; luca.marcolungo@univr.it; cristina.beltrami@univr.it; elisabetta.fortunati@univr.it; marzia.rossato@univr.it; massimo.delledonne@univr.it

[2] Personal Genomics s.r.l, Via Roveggia 43B, 37136, Verona, Italy; luciano.xumerle@personalgenomics.it

[3] Pediatric Neurology, Neurogenetics and Neurobiology Unit and Laboratories, Department of Neuroscience, A. Meyer Children's Hospital, University of Florence, viale Pieraccini 24, 50139 Florence, Italy; davide.mei@meyer.it; annalisa.vetro@meyer.it; renzo.guerrini@meyer.it; elena.parrini@meyer.it

\+ B.I. and L.X. contributed equally.

* Correspondence: massimo.delledonne@univr.it; Tel.: +39 045 8027962

Supplementary Figure S1. Increase in the genotypability of RefSeq genes for Twist using different DNA fragment lengths.

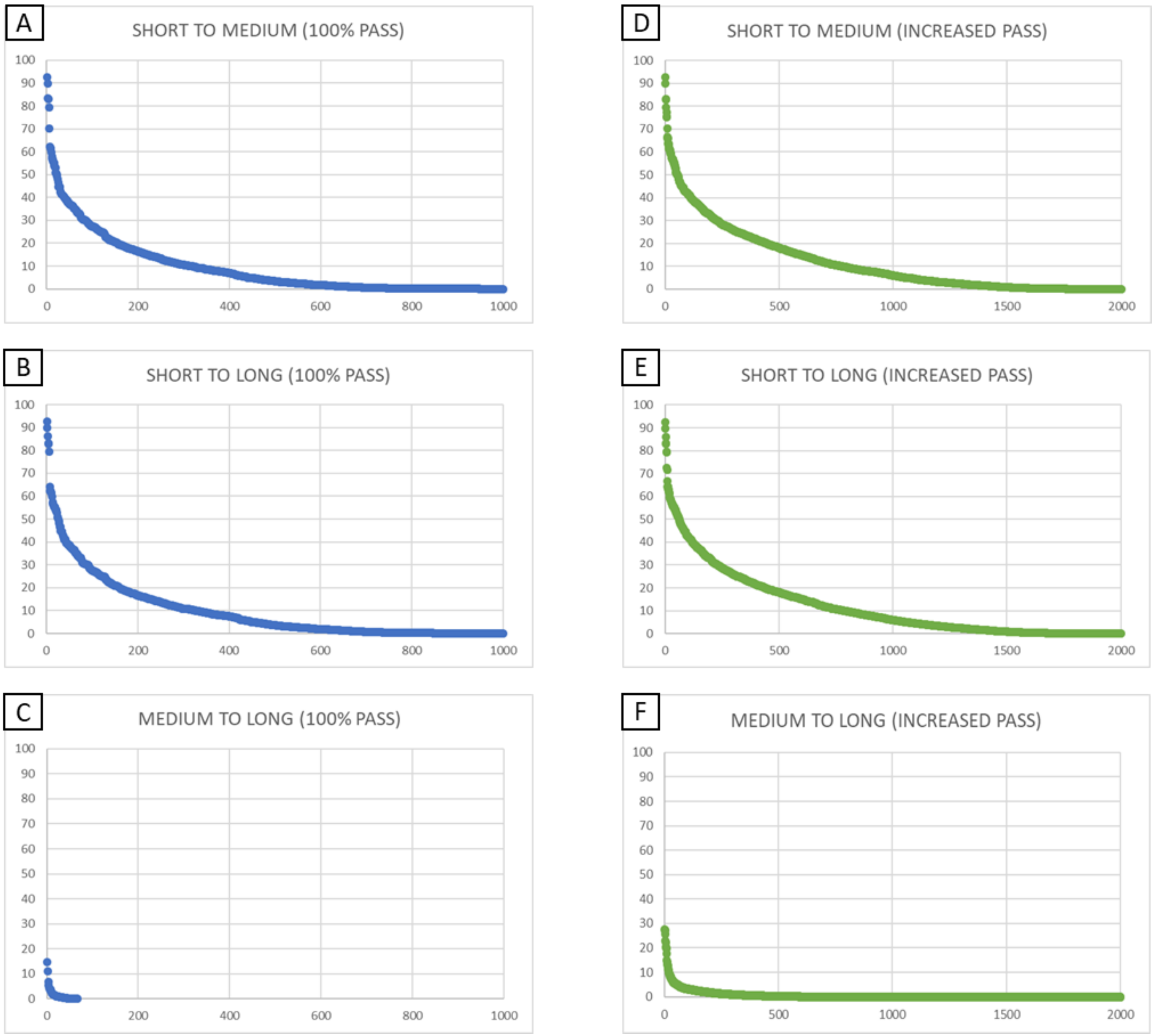


*The percentage increase in genotypability of RefSeq genes at 80X mapped coverage is shown on the Twist design, considering the DNA fragment extension from short to medium* ***(A-D)****, from short to long* ***(B-E)*** *and from medium to long* ***(C-F)*** *for the genes that reached 100% genotypability* ***(A-B-C)*** *and those showing any increase in genotypability due to an extension of the DNA fragment size* ***(D-E-F)****.*

Supplementary Figure S2. Increase in the genotypability of OMIM genes for Twist using different DNA fragment lengths.

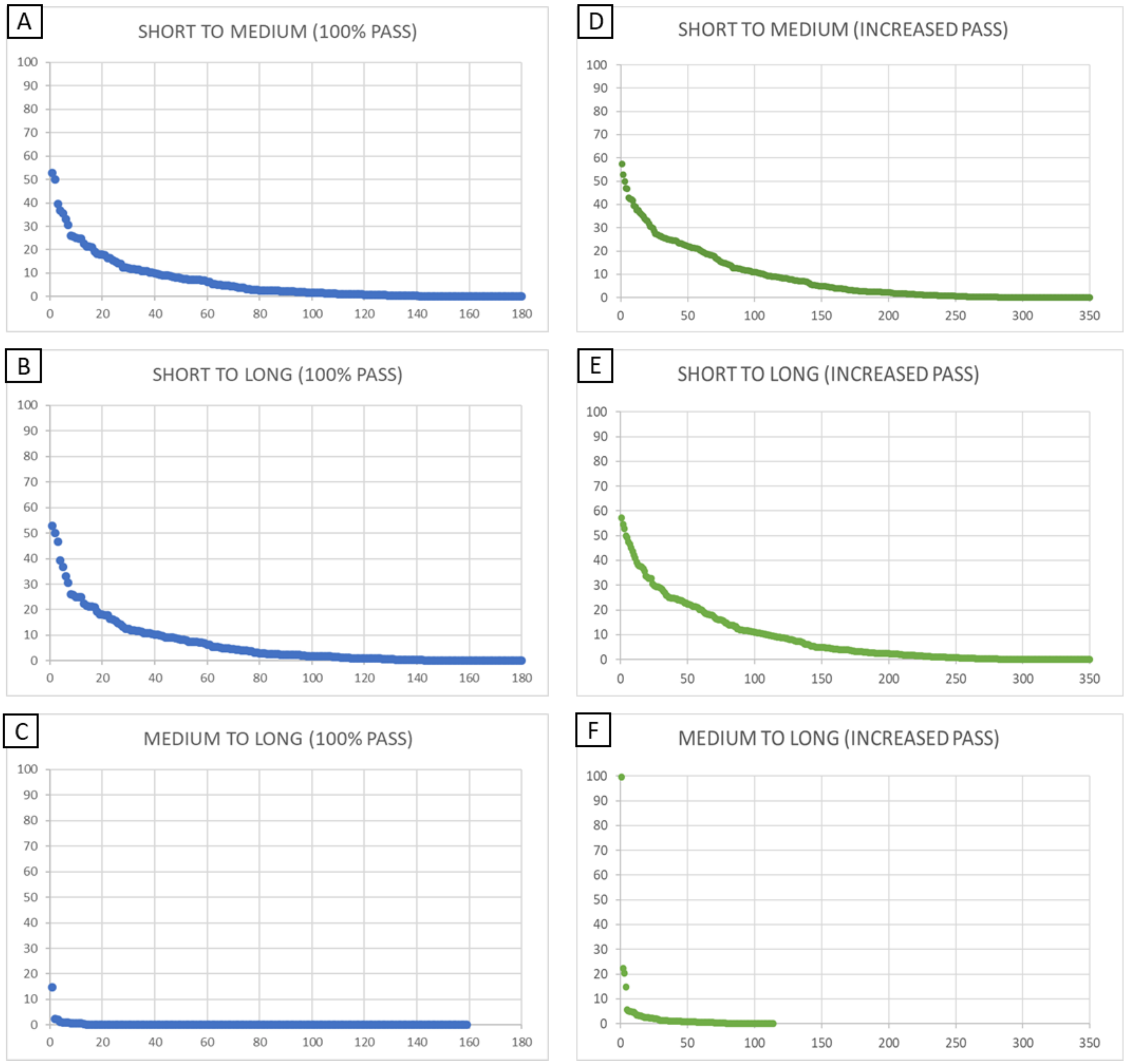


*The percentage increase in genotypability of OMIM genes at 80X mapped coverage is shown on the Twist design, considering the DNA fragment extension from short to medium* ***(A-D)****, from short to long* ***(B-E)*** *and from medium to long* ***(C-F)*** *for the genes that reached 100% genotypability* ***(A-B-C)*** *and those showing any increase in genotypability due to an extension of the DNA fragment size* ***(D-E-F)****.*

Supplementary Figure S3. Drop in genotypability of first exon of MVB12B gene from medium to long DNA fragment length.

**MVB12B: chr9:126326929-126327010**

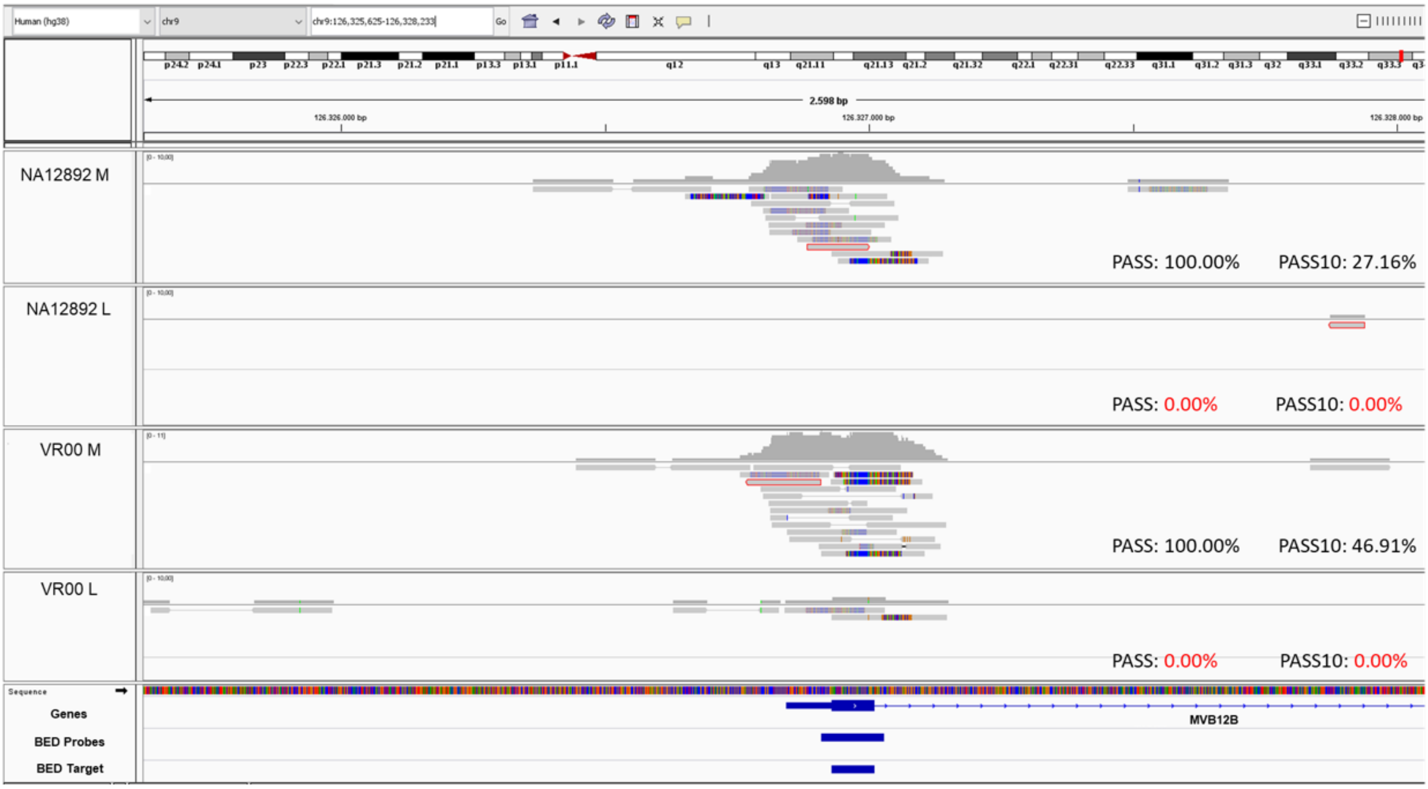


*Genotypability (PASS and PASS10 values) of the first exon of MVB12B gene in the NA12892 and VR00 samples using medium-size DNA fragment (above) and long-size DNA fragment (below). The BAM files of the samples are shown on the Twist design at 80X mapped coverage.*

Supplementary Table S1. WES initial dataset.

| ID | Sequenced fragments | GC% | Design length | Theoretical coverage (X) | Mapped deduplicated fragments | Average insert size | % Duplicates | Mapped coverage (X) |
|---|---|---|---|---|---|---|---|---|
| NA12891_IDT-S | 41,735,851 | 52 | 38,871,205 | 161.05 | 34,798,458 | 170.56 | 14.76 | 78.02 |
| NA12891_IDT-M | 31,721,147 | 51 | 38,871,205 | 244.82 | 27,295,886 | 338.36 | 12.57 | 95.92 |
| NA12891_IDT-L | 35,056,454 | 52 | 38,871,205 | 270.56 | 29,048,970 | 419.16 | 15.62 | 97.28 |
| NA12891_Roche-S | 69,139,041 | 48 | 47,007,710 | 220.62 | 53,971,159 | 250.20 | 19.09 | 92.04 |
| NA12891_Roche-M | 30,139,859 | 48 | 47,007,710 | 192.35 | 25,187,948 | 352.58 | 14.76 | 73.15 |
| NA12891_Roche-L | 37,597,648 | 48 | 47,007,710 | 239.95 | 31,408,182 | 475.51 | 14.05 | 81.22 |
| NA12891_Agilent-S | 69,997,150 | 51 | 60,448,148 | 173.70 | 56,394,632 | 267.56 | 16.11 | 85.70 |
| NA12891_Agilent-M | 43,377,376 | 50 | 60,448,148 | 215.28 | 36,041,040 | 350.30 | 15.74 | 96.23 |
| NA12891_Agilent-L | 62,476,876 | 49 | 60,448,148 | 310.07 | 50,712,887 | 438.63 | 15.70 | 122.77 |
| NA12891_Twist-S | 62,145,209 | 52 | 36,715,240 | 253.89 | 53,990,842 | 211.43 | 9.24 | 106.91 |
| NA12891_Twist-M | 45,101,242 | 49 | 36,715,240 | 368.52 | 37,418,907 | 390.49 | 14.57 | 106.47 |
| NA12891_Twist-L | 56,814,853 | 49 | 36,715,240 | 464.23 | 48,349,215 | 389.09 | 12.24 | 136.39 |
| NA12892_IDT-S | 39,279,677 | 52 | 38,871,205 | 151.58 | 33,418,710 | 174.23 | 12.97 | 74.92 |
| NA12892_IDT-M | 29,737,235 | 51 | 38,871,205 | 229.51 | 25,829,978 | 340.75 | 11.40 | 90.66 |
| NA12892_IDT-L | 31,442,649 | 52 | 38,871,205 | 242.67 | 26,557,527 | 422.28 | 13.69 | 88.66 |
| NA12892_Roche-S | 63,475,661 | 48 | 47,007,710 | 202.55 | 51,637,279 | 263.49 | 15.33 | 86.49 |
| NA12892_Roche-M | 25,113,466 | 48 | 47,007,710 | 160.27 | 21,737,831 | 353.95 | 11.70 | 63.42 |
| NA12892_Roche-L | 35,647,295 | 48 | 47,007,710 | 227.50 | 29,755,564 | 483.46 | 13.90 | 76.69 |
| NA12892_Agilent-S | 63,367,878 | 50 | 60,448,148 | 157.25 | 50,458,064 | 270.99 | 16.64 | 76.08 |
| NA12892_Agilent-M | 38,379,719 | 50 | 60,448,148 | 190.48 | 32,736,504 | 357.00 | 13.32 | 87.30 |
| NA12892_Agilent-L | 59,863,365 | 49 | 60,448,148 | 297.10 | 49,779,709 | 446.38 | 13.80 | 117.87 |
| NA12892_Twist-S | 58,556,655 | 52 | 36,715,240 | 239.23 | 51,774,402 | 207.55 | 7.76 | 102.74 |
| NA12892_Twist-M | 53,142,446 | 49 | 36,715,240 | 434.23 | 44,835,844 | 360.36 | 13.57 | 131.09 |
| NA12892_Twist-L | 53,633,893 | 49 | 36,715,240 | 438.24 | 46,784,910 | 411.80 | 9.39 | 130.16 |
| VR00_IDT-S | 43,858,514 | 52 | 38,871,205 | 169.25 | 36,430,811 | 170.05 | 14.88 | 81.28 |
| VR00_IDT-M | 30,647,544 | 51 | 38,871,205 | 236.53 | 26,574,858 | 343.45 | 11.82 | 92.66 |
| VR00_IDT-L | 28,719,060 | 51 | 38,871,205 | 221.65 | 24,489,849 | 429.35 | 13.02 | 80.87 |
| VR00_Roche-S | 69,341,641 | 47 | 47,007,710 | 221.27 | 55,349,772 | 262.24 | 16.77 | 80.89 |
| VR00_Roche-M | 25,769,903 | 48 | 47,007,710 | 164.46 | 22,210,538 | 361.45 | 11.86 | 64.00 |
| VR00_Roche-L | 37,393,464 | 47 | 47,007,710 | 238.64 | 31,375,345 | 482.07 | 13.60 | 80.81 |
| VR00_Agilent-S | 63,423,698 | 51 | 60,448,148 | 157.38 | 51,058,660 | 265.11 | 17.83 | 77.59 |
| VR00_Agilent-M | 37,984,977 | 50 | 60,448,148 | 188.52 | 31,756,515 | 354.10 | 15.15 | 84.83 |
| VR00_Agilent-L | 34,450,041 | 49 | 60,448,148 | 170.97 | 28,964,914 | 439.13 | 13.13 | 69.93 |
| VR00_Twist-S | 58,092,508 | 52 | 36,715,240 | 237.34 | 51,158,376 | 209.79 | 8.01 | 101.11 |
| VR00_Twist-M | 49,286,947 | 49 | 36,715,240 | 402.72 | 42,067,556 | 360.41 | 12.36 | 121.92 |
| VR00_Twist-L | 53,025,771 | 48 | 36,715,240 | 433.27 | 46,216,132 | 400.91 | 9.92 | 128.25 |

*For each replicate, platform and DNA fragment length combination, the number of sequenced fragments, percentage GC content, theoretical coverage, number of mapped fragments without duplicates, average insert size, percentage of reads marked as duplicates and mapped coverage on the target are shown. DNA fragment lengths: S = short, M = medium, L = long.*

Supplementary Table S2. The 200X dataset.

| DNA fragment length | Average insert size | % Duplicates | Mapped coverage (X) | %1X | %5X | %10X | %20X | %30X | % PASS | % PASS RD>10 | % ON TARGET | % NEAR TARGET | % OFF TARGET | Fold enrichment | FOLD 80 penalty |
|---|---|---|---|---|---|---|---|---|---|---|---|---|---|---|---|
| 200 | 206.28 | 6.29 | 87.30 | 99.86 | 99.82 | 99.70 | 98.36 | 95.22 | 95.50 | 95.39 | 52.36 | 32.96 | 14.68 | 45.89 | 1.58 |
| 230 | 231.64 | 9.24 | 80.90 | 99.86 | 99.82 | 99.71 | 98.40 | 95.17 | 95.53 | 95.43 | 49.78 | 35.58 | 14.63 | 43.63 | 1.53 |
| 260 | 262.34 | 5.31 | 61.15 | 99.80 | 99.72 | 99.64 | 99.32 | 97.15 | 96.38 | 96.33 | 40.20 | 36.52 | 23.28 | 35.23 | 1.36 |
| 270 | 270.91 | 5.08 | 61.86 | 99.78 | 99.71 | 99.64 | 99.34 | 97.33 | 96.41 | 96.36 | 39.73 | 37.21 | 23.06 | 34.82 | 1.36 |
| 280 | 281.83 | 5.11 | 61.81 | 99.78 | 99.69 | 99.61 | 99.25 | 97.03 | 96.42 | 96.35 | 38.70 | 37.43 | 23.87 | 33.92 | 1.38 |
| 290 | 290.87 | 5.32 | 61.37 | 99.79 | 99.69 | 99.61 | 99.24 | 96.87 | 96.43 | 96.35 | 37.89 | 38.05 | 24.06 | 33.21 | 1.38 |
| 340 | 334.18 | 10.16 | 57.26 | 99.83 | 99.74 | 99.63 | 98.96 | 94.90 | 96.54 | 96.44 | 36.35 | 40.44 | 23.20 | 31.86 | 1.38 |
| 360 | 366.62 | 7.18 | 64.74 | 99.83 | 99.76 | 99.60 | 98.16 | 94.11 | 96.58 | 96.43 | 38.80 | 45.65 | 15.56 | 34.00 | 1.44 |
| 400 | 396.33 | 4.94 | 63.50 | 99.83 | 99.75 | 99.56 | 97.78 | 93.14 | 96.59 | 96.39 | 37.16 | 47.03 | 15.81 | 32.57 | 1.48 |

*For each DNA fragment length, the 200 theoretical X-fold coverage is shown for the Twist target design dataset (mean of three independent experiments). The columns show the average insert size, percentage of reads marked as duplicates, mapped coverage on the target, percentage of the target covered by at least 1, 5, 10, 20 and 30 reads, percentage of callable bases on the target for standard read depth (>3) and read depth >10, percentage of bases on/near/off target, fold enrichment and FOLD 80 base penalty.*

Supplementary Table S3. Genotypability of RPL15, RPS26, RPSA and PTEN genes.

| Platform | Short (% PASS) | Medium (% PASS) | Long (% PASS) | % Diff. |
|---|---|---|---|---|
| **RPL15** | | | | |
| IDT | 34.49 | 88.74 | 88.74 | 54.25 |
| Roche | 84.90 | 100.00 | 100.00 | 15.10 |
| Agilent | 83.50 | 100.00 | 100.00 | 16.50 |
| Twist | 49.98 | 100.00 | 100.00 | 50.02 |
| **RPS26** | | | | |
| IDT | 69.35 | 100.00 | 100.00 | 30.65 |
| Roche | 92.05 | 100.00 | 100.00 | 7.95 |
| Agilent | 93.39 | 100.00 | 100.00 | 6.61 |
| Twist | 47.13 | 100.00 | 100.00 | 52.87 |
| **RPSA** | | | | |
| IDT | 69.45 | 100.00 | 100.00 | 30.55 |
| Roche | 96.25 | 100.00 | 100.00 | 3.75 |
| Agilent | 95.46 | 100.00 | 100.00 | 4.54 |
| Twist | 63.29 | 100.00 | 100.00 | 36.71 |
| **PTEN** | | | | |
| IDT | 77.58 | 100.00 | 100.00 | 22.42 |
| Roche | 84.63 | 100.00 | 100.00 | 15.37 |
| Agilent | 90.89 | 100.00 | 100.00 | 9.11 |
| Twist | 78.55 | 100.00 | 100.00 | 21.45 |

Supplementary Table S4. Library preparation specifications for each enrichment platform.

| ENRICHMENT PLATFORM | INPUT (ng) | INSERT SIZE (bp) | DNA shearing | | AMPure size selection | PCR cycles |
|---|---|---|---|---|---|---|
| | | | COVARIS treatment time (sec) | Enzymatic digestion time (min) | | |
| IDT | 100 | 150 | 375 | | | 10 |
| | | 350 | 110 | | 0.75X | 10 |
| | | 500 | 85 | | 0.60X | 12 |
| ROCHE | 500 | 200 | 235 | | | 5 |
| | | 350 | 110 | | 0.75X | 5 |
| | | 500 | 85 | | 0.60X | 7 |
| AGILENT | 1500 | 200 | 235 | | | 6 |
| | | 350 | 110 | | 0.75X | 6 |
| | | 500 | 85 | | 0.60X | 8 |
| TWIST | 50 | 200 | | 22 | | 10 |
| | | 350 | 110 | | 0.7X | 10 |
| | | 500 | 85 | | 0.65X | 10 |

Supplementary Table S5. Library preparation specifications for the 27 individuals selected from our internal database (Twist platform).

| **ENRICHMENT PLATFORM** | **INPUT (ng)** | **INSERT SIZE (bp)** | **DNA shearing Enzymatic digestion time (min)** | **AMPure size selection** | **PCR cycles** |
|---|---|---|---|---|---|
| TWIST | 50 | 200-400 | 16.30 | 0.7X | 10 |
